# Recalibration and Validation of a Risk Scoring Tool to Predict Multidrug-Resistant *Pseudomonas aeruginosa*

**DOI:** 10.1093/ofid/ofag359

**Published:** 2026-06-17

**Authors:** Nhu Le, Hyunuk Seung, Megan E Dunning, Emily L Heil, Kimberly C Claeys

**Affiliations:** University of Maryland School of Medicine, Baltimore, Maryland, USA; Department of Pharmacy Practice, Sciences, and Health Outcomes Research, University of Maryland School of Pharmacy, Baltimore, Maryland, USA; Division of Infectious Diseases, Department of Medicine, University of Maryland School of Medicine, Baltimore, Maryland, USA; Department of Pharmacy Practice, Sciences, and Health Outcomes Research, University of Maryland School of Pharmacy, Baltimore, Maryland, USA; Department of Pharmacy Practice, Sciences, and Health Outcomes Research, University of Maryland School of Pharmacy, Baltimore, Maryland, USA

**Keywords:** antimicrobial resistance, difficult-to-treat resistance (DTR), model calibration and validation, *Pseudomonas aeruginosa*

## Abstract

We recalibrated a risk score to identify difficult-to-treat resistant *Pseudomonas aeruginosa* among 197 intensive care unit patients with bloodstream or respiratory infection. Prevalence was 6%. Models showed moderate discrimination (c-statistic 0.71–0.74), low sensitivity (18%), and high specificity (94%–96%), highlighting the need for local validation and consideration of performance tradeoffs.

*Pseudomonas aeruginosa* is a public health threat due to the limited number of therapeutic options, which are made more scarce in the presence of numerous intrinsic and acquired resistance mechanisms [1]. This is underscored by the Infectious Diseases Society of America (IDSA) guidance, which proposed the clinically relevant and contemporary “difficult-to-treat resistance” (DTR) definition to better reflect current therapeutic decision-making [1]. Resistance to carbapenems for *P aeruginosa* has been reported to be upwards of 30% and DTR reported as approximately 8% in the United States [2, 3]. This makes ensuring timely and effective antimicrobial therapy extremely challenging.

A major obstacle to initiating active and targeted antimicrobial therapy is the lack of rapid diagnostics that can detect resistance mechanisms common to *P aeruginosa* [4, 5]. This can lead to days of delays in identifying phenotypic resistance, with outcomes becoming increasingly worse with delays in receipt of in vitro active therapy [6, 7]. A recent study of patients with bloodstream infections reported that the odds of 30-day mortality increased with every day of delayed active therapy [6]. This is of particular concern for DTR *P aeruginosa*, as therapeutic options like ceftolozane-tazobactam often have reports of phenotypic susceptibility substantially delayed due to lack of readily available automated testing [7]. This is why numerous clinical prediction models have been developed and published [8–12]. However, data supporting their use outside derivation cohorts, including local validation and recalibration, remain limited.

In 2023, we published a simplified risk scoring tool to predict multidrug resistance among respiratory or bloodstream infections caused by *P aeruginosa* (defined in the study as nonsusceptible to at least 2 of 3 antimicrobial agents: cefepime, piperacillin-tazobactam, and/or meropenem) [10]. Using the individual risk factors from previously published models, a new, parsimonious model was created with 4 readily retrievable clinical variables: prior multidrug-resistant (MDR) *P aeruginosa*, ≥4 antimicrobials before culture during hospitalization, infection onset >3 days after admission (not present on admission), and ongoing hemodialysis. To align with contemporary national guidance and address the limited reporting on calibration and clinical usability of resistance prediction models, we sought to update an existing risk score to predict DTR *P aeruginosa* and evaluate its performance using contemporary institutional data. Therefore, before implementing the novel risk scoring tool within our local electronic medical record (EMR), we wished to (1) update the definition to DTR *P aeruginosa* and (2) calibrate the model to optimize clinical utility [13].

## METHODS

We conducted a retrospective, single-center cohort analysis to recalibrate a previously published simplified risk score and adjusted to predicting DTR *P aeruginosa* from a previous internal definition of MDR *P aeruginosa*. This study included adult (age ≥18 years) intensive care unit (ICU) patients with *P aeruginosa* infection admitted to the University of Maryland Medical Center, a tertiary care center, between January 2021 and March 2024. Eligible patients had at least 1 clinical blood or respiratory culture positive for *P aeruginosa* with available phenotypic susceptibility data and received empiric antimicrobial therapy directed toward the *P aeruginosa* infection. Patients were evaluated using both the original multidrug resistance definition applied in the derivation study and the updated IDSA definition for DTR *P aeruginosa* [13]. This study was institutional review board approved (HP-00109236) with a waiver of informed consent.

After eligible patients were identified, information collected from the EMR comprised age, type of ICU, site of *P aeruginosa* infection, admission duration >72 hours, previous antibiotics, pertinent past medical history including respiratory comorbidities and immunocompromising conditions, previous hospitalizations within 1 year, and previous isolation of clinical or surveillance MDR gram-negative organisms within 1 year. Both the original multidrug resistance outcome used in the derivation study and the updated IDSA definition of DTR *P aeruginosa* were applied. The previously identified candidate predictors (prior MDR *P aeruginosa* or MDR gram-negative organism, exposure to ≥4 antibiotics during hospitalization before culture, infection not present on admission, and inpatient hemodialysis) were reassessed using contemporary institutional data. Initial comparisons between patients with and without DTR *P aeruginosa* were completed using Fisher exact test or χ^2^ test for discrete variables and Student *t*-test or Mann–Whitney *U* test for continuous variables.

The primary outcome was detection of DTR among those with *P aeruginosa* bloodstream or respiratory tract infection. Multiple parsimonious model variants were evaluated using different combinations of these prespecified predictors to assess robustness and clinical usability. Logistic regression was used for model fitting, and Firth penalized likelihood estimation was applied to mitigate small-sample bias and separation due to the low prevalence of DTR events. Regression coefficients from each fitted model were transformed into weighted integer values proportional to their effect sizes to construct simplified risk scoring tools suitable for bedside or electronic implementation.

Model discrimination was assessed using the area under the receiver operating characteristic (ROC) curve. Clinically relevant score cutoffs were selected using the closest-to-(0,1) method on the ROC curve to balance sensitivity and specificity. Model calibration was evaluated by comparing predicted and observed event rates across deciles of predicted risk and visualized using calibration plots. Predicted probabilities were recalibrated using isotonic regression to improve agreement between predicted and observed event rates. A *P* value of <.05 was considered statistically significant and all analyses were 2-tailed. Analyses were performed using SAS version 9.4 software (SAS Institute, Cary, NC, USA).

## RESULTS

A total of 197 adult ICU patients with bloodstream or respiratory *P aeruginosa* infections were included. The median age was 55 years and 132 (67%) were male. The most common source of infection was respiratory hospital-acquired bacterial pneumonia/ventilator-associated pneumonia (n = 85 [43%]). Among this updated cohort, MDR *P aeruginosa* was identified in 27 (14%) patients, and DTR *P aeruginosa* identified in 11 (6%) patients. Several risk factors included in the previously published score were more frequently observed among patients with DTR infection, including receipt of ≥4 prior antibiotics and inpatient hemodialysis; however, the low number of DTR events limited statistical precision (Table 1).

**Table 1.** Comparison and Associated *P* values for 4 Risk Factors Between Patients With Non–Difficult-to-Treat Resistance (DTR) Versus DTR *Pseudomonas aeruginosa*

Risk Factor	Non-DTR *Pa* (n = 186)	DTR *Pa* (n = 11)	*P* Value
Previous MDR *Pa* culture (6 mo)	17 (9%)	1 (5%)	.4
Receiving dialysis (any form)	120 (64%)	3 (28%)	.08
Not infected upon admission (>72 h)	17 (9%)	3 (26.2%)	.3
Received ≥4 previous antibiotics during admission	135 (72%)	4 (35.3%)	.02

Abbreviations: DTR, difficult-to-treat resistance; MDR, multidrug-resistant; *Pa*, *Pseudomonas aeruginosa*.

Three simplified risk scoring models using different combinations of prespecified clinical predictors were evaluated (Table 2). The previous study sample, with updated DTR definition, was used as a training cohort (n = 280, events = 20) while the new sample was used as the test cohort (n = 198, events = 11) to develop new, calibrated models to predict DTR *P aeruginosa*. Across all models, sensitivity for predicting DTR *P aeruginosa* was low (18%), reflecting the infrequent occurrence of the outcome. Specificity was consistently high, ranging from 94% to 96%. Discrimination in the test dataset was moderate, with the c-statistic ranging from 0.71 to 0.74.

**Table 2.** Simplified Risk Score (Derivation) and Model Performance (Test Cohort)

Risk Score and Model Performance	Model 1	Model 2	Model 3
Risk factor (score)	≥4 Abx (1) Not POA (1) Prior MDRGN (2)	≥4 Abx (4) Prior MDRGN (6) Inpatient dialysis (1)	≥4 Abx (1) Prior MDRGN (2)
Risk score range	0–4	0–11	0–3
Risk score cutoff	2	4	1
Model performance (test cohort)			
Accuracy, c-statistic (95% CI)	0.71 (.55–.88)	0.74 (.58–.90)	0.71 (.56–.86)
Sensitivity	18.2%	18.2%	18.2%
Specificity	89.9%	91.4%	91.9%

Risk scores were derived from the training cohort; model performance was evaluated in the test cohort.

Abbreviations: Abx, antibiotics; CI, confidence interval; MDRGN, multidrug-resistant gram-negative organism; POA, present on admission.

**Figure 1.**
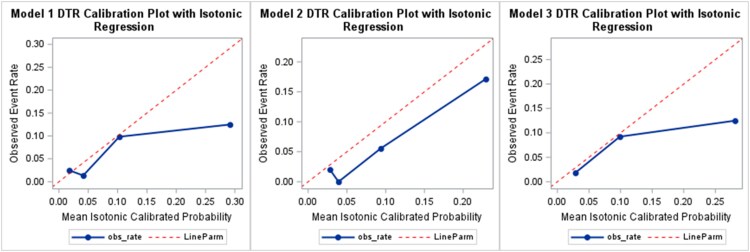
Calibration plots for model predictions of *Pseudomonas aeruginosa* with difficult-to-treat resistance. Abbreviations: DTR, difficult-to-treat resistance; LineParm, ideal calibration line or line of perfect agreement; obs_rate, observed event rate.

Calibration curves (Figure 1) demonstrated variable agreement between predicted and observed DTR event rates. Among evaluated models, model 2 showed the closest alignment between predicted and observed probabilities in the test cohort, balancing high specificity (95.7%) with stable discrimination, making it the best score for a goal of screening high-risk patients for urgent targeted therapy with minimal false positives. However, some degree of miscalibration remained across models, and additional validation with larger patient datasets that contain more DTR-positive cases is needed to fully refine the model before implementation.

## DISCUSSION

In this report, we describe the local recalibration and performance of a previously published simplified risk scoring tool for DTR *P aeruginosa* using contemporary institutional data. Consistent with previously published work, DTR infection was uncommon in this population, occurring in approximately 6% of critically ill patients with bloodstream or respiratory *P aeruginosa* [3]. This low event prevalence substantially influenced model performance and underscored important tradeoffs relevant to clinical implementation.

Calibration analyses further demonstrated the importance of institutional validation before clinical adoption of risk scoring tools to predict drug resistance. Among the evaluated models, 1 configuration demonstrated the most consistent alignment between predicted and observed DTR event rates, supporting its relative suitability for local use. However, calibration remained imperfect, highlighting that performance characteristics observed in derivation cohorts cannot be assumed to generalize across settings or time periods without recalibration. Although most models undergo validation through a separate sample, they are often not accompanied by considerations for calibration, decision-analytic measures, or acceptability [14, 15]. Calibration is a critical element to ensure reliable model performance across diverse populations; as highlighted by Van Calster et al, poorly calibrated models may mislead clinical decisions and ultimately cause patient harm [16].

Several limitations of this study warrant consideration. This analysis was conducted at a single center and was constrained by a small number of DTR events, limiting precision and generalizability of the results. In addition, the retrospective design precludes assessment of real-time clinical impact. Importantly, this tool is intended for use only among patients with confirmed *P aeruginosa*, such as those identified through rapid diagnostic testing, and is not designed for empiric screening of undifferentiated infections. Last, we have not addressed the challenges of how to integrate this tool into the EMR to aid in pragmatic decision-making.

Despite these limitations, our findings provide practical insight into the challenges of translating resistance prediction models from publication into practice. Local recalibration proved essential for understanding model performance and defining an implementation strategy aligned with institutional priorities. Future work will focus on validation in larger, more diverse populations and integration into EMR clinical decision support systems, enabling prospective evaluation of effects on antibiotic selection, stewardship outcomes, and patient-centered endpoints.
